# Influence of Organisational-Level Factors on Delayed Door-to-Balloon Time among Patients with ST-Elevation Myocardial Infarction

**DOI:** 10.18295/squmj.5.2023.038

**Published:** 2024-05-27

**Authors:** Munira A. Al-Rumhi, Sulaiman D. Al Sabei, Huda S. Al-Noumani, Adil Al-Riyami, Omar Al-Rawajfah

**Affiliations:** 1 Emergency Department, Sultan Qaboos University Hospital, Muscat, Oman; 3 Department of Medicine, Sultan Qaboos University Hospital, Muscat, Oman; 2 Department of Nursing, Sultan Qaboos University, Muscat, Oman; 4 Department of Nursing, Al-Bayt University, Al-Mafraq, Jordan

**Keywords:** Acute Myocardial Infarction, Coronary Balloon Angioplasty, Patient Care Management, Emergency Care Systems, Staffing and Scheduling, Oman

## Abstract

**Objectives:**

This study aimed to estimate the door-to-balloon (DTB) time and determine the organisational-level factors that influence delayed DTB times among patients with ST-elevation myocardial infarction in Oman.

**Methods:**

A cross-sectional retrospective study was conducted on all patients who presented to the emergency department at Sultan Qaboos University Hospital and Royal Hospital, Muscat, Oman, and underwent primary percutaneous coronary interventions during 2018–2019.

**Results:**

The sample included 426 patients and the median DTB time was 142 minutes. The result of the bivariate logistic regression showed that patients who presented to the emergency department with atypical symptoms were 3 times more likely to have a delayed DTB time, when compared to patients who presented with typical symptoms (odds ratio [OR] = 3.003, 95% confidence interval [CI]: 1.409–6.400; *P* = 0.004). In addition, patients who presented during off-hours were 2 times more likely to have a delayed DTB time, when compared to patients who presented during regular working hours (OR = 2.291, 95% CI: 1.284–4.087; *P* = 0.005).

**Conclusion:**

To meet the DTB time recommendation, it is important to ensure adequate staffing during both regular and irregular working hours. Results from this study can be used as a baseline for future studies and inform strategies for improving the quality of care.

**Advances in Knowledge**

- *The time of the day patients presented was significantly associated with delayed door-to-balloon (DTB) times.*
- *The inability of triage nurses to recognise the symptoms of patients with ST-elevation myocardial infarction was associated with delayed DTB times.*
- *Approximately 90% of the females who presented with atypical symptoms experienced a delay in DTB times.*

**Applications to Patient Care**

- *Training nurses in the emergency triage rooms to identify patients with ST-elevation myocardial infarction and ensuring adequate staffing during both regular and irregular working hours are critical in performing timely surgical interventions for patients with STelevated myocardial infarction.*

Heart disease is the number one cause of death globally. According to the latest statistics released by the American Heart Association (AHA), approximately 18.6 million deaths occurred globally from heart disease in 2019.^1^ One of the most common heart diseases is ST-segment elevation myocardial infarction (STEMI). STEMI has been associated with negative healthcare outcomes including prolonged length of hospitalisation and increased in-hospital mortality.^2^^,^^3^ Therefore, treating STEMI is one of the top priorities of healthcare institutions. Performing timely primary percutaneous coronary interventions (PPCIs) is critical in saving the lives of patients with STEMI.^4^ The door-to-balloon (DTB) time is one of the quality indicators for performing timely PPCIs.^5^

The DTB time refers to the time between a patient’s arrival at the hospital and the inflation of the first balloon or device.^5^ The DTB time should last no more than 90 minutes, according to the most recent guidelines published by the AHA and the American College of Cardiology (ACC).^5^ Patients with delayed DTB times have poorer outcomes such as impaired left ventricular ejection fraction, prolonged hospitalisation and higher crude in-hospital mortality.^3^^,^^6^

Investigating the factors contributing to the delayed DTB time is critical in ensuring safe practices. Studies showed that many factors influence DTB times, including the gender of the patient, presenting time and presenting symptoms.^7^–^9^ The main gap identified in the literature is that most studies assessing the factors responsible for delayed DTB times were conducted in Western countries. Studies conducted in Eastern countries, including Oman, in this regard are limited in number. The healthcare system in Western countries is different from that of Eastern countries, which may suggest that different factors can be associated with DTB time in Eastern countries, depending on the context.

This study aimed to estimate the DTB time and determine the organisational-level factors that influence delayed DTB times among patients with ST-elevation myocardial infarction (MI) in Oman.

## Methods

This retrospective cross-sectional study was conducted at the Royal Hospital and Sultan Qaboos University Hospital (SQUH), Muscat, Oman, and included data from patients who had undergone PPCI over two years (from January 2018 to December 2019). The PCI registry is a prospective registry designed to collect and record PCI types and their timings. The PPCIs were extracted, and the list was generated. The principal investigator reviewed and scanned the medical records of the listed patients. This task involved reviewing nursing notes, physician notes and laboratory results. The catheterisation (balloon) times were extracted from the catheterisation laboratory (cath lab) registry, which is available in both hospitals. The study included patients aged 18 years or older who presented with STEMI. Patients who underwent PPCI more than once during the study period were counted as new cases each time. Referred STEMI patients who were managed from other healthcare facilities, patients who did not choose PPCI as their primary reperfusion therapy and patients who underwent elective PCI were excluded from the study.

The dependent variable, DTB time, was measured from the time of registration in the emergency department (ED) to the time of intervention as recorded in the patient’s health record system. A DTB time of more than 90 minutes was considered a delayed time.

The independent variables were age, gender, troponin level, history of comorbidities (diabetes, hypertension and dyslipidaemia), previous MI, previous coronary artery bypass graft (CABG), smoking status, presenting time (off-hours versus regular working hours), triage level haemodynamic status (low versus severe), presenting symptoms (atypical versus typical) and presenting status (stable versus non-stable). Patients with STEMI who presented to the ED without congestive heart failure, hypotension or cardiac arrhythmia were considered stable.^10^ Patients who presented with chest, arm, jaw and radiating symptoms such as nausea, vomiting, sweating, dyspnoea and palpitation were classified as typical.^11^ Patients who presented to the ED with indigestion-like symptoms from the triage chief complaint episode as indicated in the medical record were classified as atypical.^11^ Accordingly, a variable called ‘presenting symptoms’ was created in Statistical Package for Social Sciences (SPSS), Version 23 (IBM Corp., Armonk, New York, USA). The variable was dichotomous and responses were either typical or atypical.

Patient presenting time was defined as the comparison of off-hours versus regular hours as weekend and night versus weekday regular hours, weekend versus weekday or night versus daytime.^12^ Operationally, patients who presented to the ED between 7 AM and 3 PM from Sunday to Thursday were recorded as patients who presented during regular working hours (Oman’s regular working days are from Sunday to Thursday), while those who presented to the ED from 3 PM to 7 AM were classified as patients presenting during off-working hours. In addition, patients who presented during weekends and public holidays were considered to have presented during off-working hours.

The data were analysed using SPSS. Statistical significance was considered at *P* <0.05. The DTB times were estimated among patients with STEMI at selected hospitals in Oman by using median and interquartile range (IQR). Mann-Whitney U test was used to compare the DTB time intervals between delayed and non-delayed DTB patients. Subsequently, the possible factors associated with delayed DTB times were examined among the same patients using bivariate logistic regression. No sensitivity analyses were conducted.

The study was approved by the Medical Research and Ethics Committee and the Ministry of Health (MREC #2140). As this was a retrospective study, no direct contact was made with the study participants and no informed consent was required. Patient confidentiality was maintained throughout the study period by removing all identification data from all study documents.

## Results

**Figure 1:**
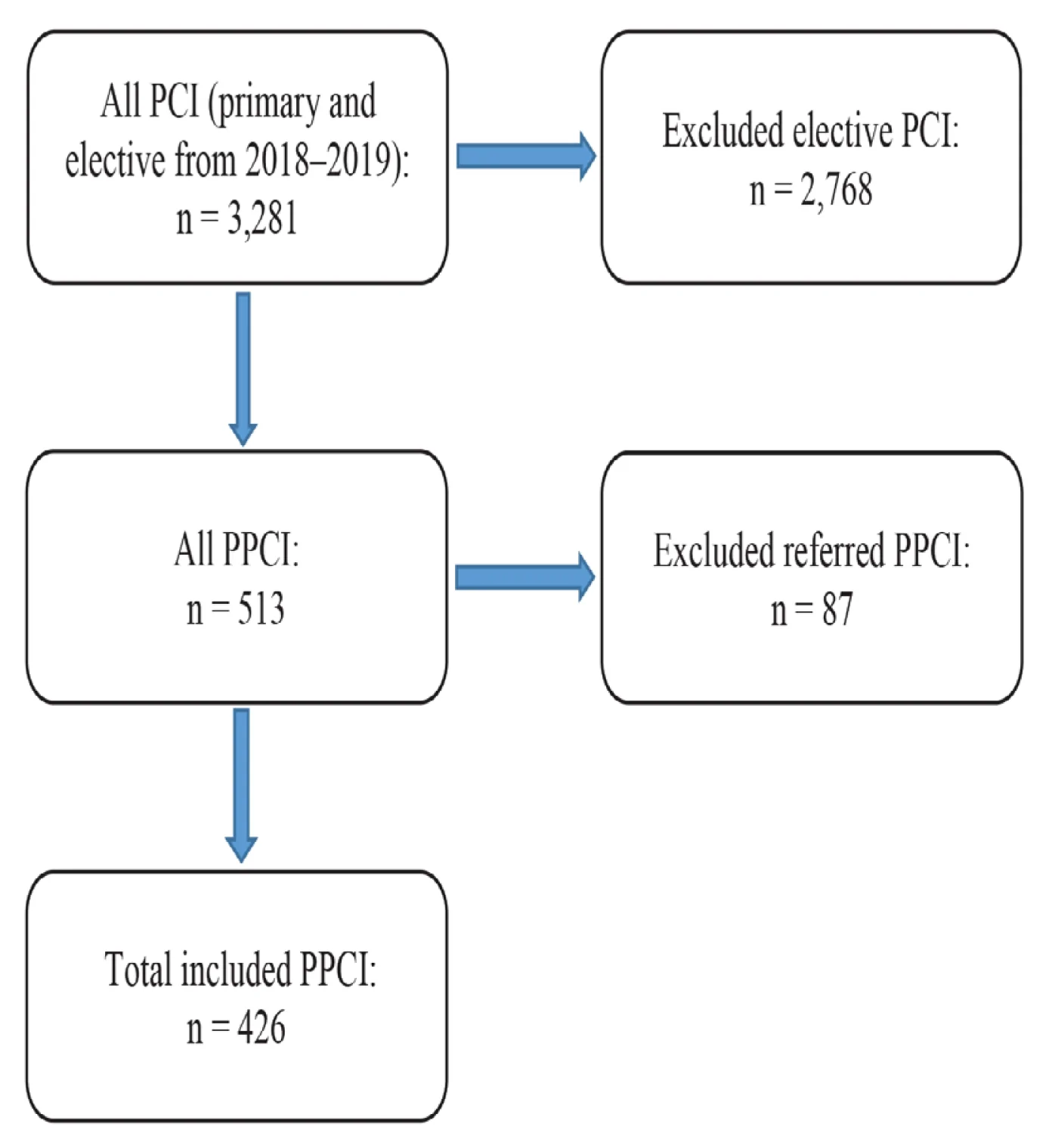
Flowchart showing patient selection criteria. *PPCI = primary percutaneous coronary intervention*.

A total of 3,281 PCIs were conducted at the participating hospitals during the study period. Of these, 2,855 cases were excluded because they were elective (n = 2,768) or referral cases (n = 87) from other hospitals that were not admitted through the ED. The final sample consisted of 426 patients (300 patients from the Royal Hospital and 126 patients from SQUH) who met the inclusion criteria [Figure 1].

The sample consisted of predominantly male (81.7%) and Omani (76.1%) patients. The mean age of the participants was 56.76 ± 12.38 years. More than half the patients (60.6%) did not have a family history of cardiac diseases. Among the patients, 54.9% (n = 234) were triaged at either level I (5.4%) or level II (49.5 %). More than three-quarters (89.9%) of the patients had positive troponin results at presentation [Table 1].

Many patients had a history of hypertension (47.7%) or diabetes (45.5%). Furthermore, 14.1% of the patients had a previous diagnosis of MI and had been treated with PCI, whereas 2.6% had undergone a CABG. Approximately 4% of the patients had a history of chronic kidney disease (CKD). In addition, 27.9% of the patients were smokers. Among the patients presenting to the ED, more than three-quarters (89.7%) were considered stable according to their vital signs. Approximately two-thirds (70.9%) presented with complaints of typical MI symptoms. The majority of study patients (76.8%) presented to the ED after official hospital hours [Table 1].

The relationship between the patients’ characteristics and DTB time was assessed using the Chi-squared test of independence for dichotomous variables, including gender and Fisher’s exact test for categorical variables such as presenting status. Findings revealed that the patients’ presenting time and symptoms were the only variables that had a statistically significant effect on DTB time (*P* = 0.005).

The median DTB time was 142 (IQR = 110–190) minutes. The majority (n = 357, 83.8%) were classified as delayed DTB times (>90 minutes), whereas only 69 cases (6.2%) had non-delayed times. Patients in the delayed DTB group spent 150 minutes from the time they reached the ED until the balloon was inflated (IQR = 128–150), while the same for the patients in the non-delayed group was 75 minutes (IQR = 67–83) [Table 2].

The overall DTB time was further divided into three intervals: 1) the average time interval spent from the door to the electrocardiogram (ECG); 2) from the ECG to arrival at the cath lab (ECG to cath lab); and 3) arrival at the cath lab to the balloon procedure [Table 2]. Findings showed that the longest time was spent between the ECG to the cath lab (median = 78 minutes, IQR = 53–98.25), followed by the time spent between the cath lab and the balloon (median = 70 minutes, IQR = 44–90.25). The time difference between the two groups was statistically significant in all DTB time-interval categories (≤0.020) [Table 2].

The DTB time intervals were also compared across the two included hospitals and showed significant differences in all DTB time intervals between the two hospitals (≤0.044) [Table 3].

Bivariate logistic regression was performed to identify factors influencing the likelihood of a delayed DTB time [Table 4]. The model was evaluated against the constant-only model and was found to be statistically significant (χ^2^ = 17.13; *P* = 0.04). The total Nagelkerke R^2^ for the model was 0.086, whereas the Cox–Snell R^2^ was 0.050.

‘Presenting symptom’ and ‘presenting time’ were the only significant factors associated with the likelihood of delayed DTB times (OR = 3.003, 95% confidence interval [CI]: 1.409–6.400; *P* = 0.004) and (OR = 2.291, 95% CI: 1.284–4.087; *P* = 0.005), respectively. The overall successful prediction rate of the model was 83.8% to classify patients in delayed and non-delayed categories. A post-estimation Hosmer-Lemeshow test was conducted to assess the goodness of fit for the logistic regression model, (χ^2^ = 10.254; *P* = 0.248).

## Discussion

The current study findings showed that 83.8% of the study patients had a delayed DTB time. The median DTB time was 142 minutes, which is longer than the time recommended by AHA and ACC for managing patients with STEMI.

At the regional level, the current study showed that the DTB times in Oman were higher than the times reported in studies conducted in Iran, Qatar, Saudi Arabia, Kuwait, Bahrain and the United Arab Emirates.^8^^,^^13^–^15^ At the international level, the DTB time in Oman was also higher than in other countries including Thailand, Singapore, Japan, Nepal, Canada, Australia and the USA.^16^–^22^

The differences in the DTB times among patients treated in Oman compared to the findings of other studies can be attributed to the differences in study design, sample size, study setting and the implementation of quality improvement projects. One of the factors that could have contributed to the variation in the reported DTB times across studies was the use of different study designs. For example, a study conducted in Nepal used a prospective design.^19^ The use of a prospective design can ensure the accuracy of data and the ability to examine many variables and results that are not possible in a retrospective approach. Moreover, sample size is another factor that can affect reported DTB times. Some studies reported a small sample size ranging from 79–150 participants.^16^^,^^19^ In addition, some studies were conducted at single centres—such as those conducted in Qatar, Iran, Saudi Arabia, Thailand, Singapore and Canada.^8^^,^^13^^,^^14^^,^^16^^,^^17^^,^^20^ The generalisability of such study findings is limited, which might be another factor that contributed to their lower DTB times compared with the current study, which collected data from two settings.

The current study found that the average time expended from the ECG to the cath lab was 85.15 ± 56.45 minutes. According to the literature, the recommended ECG-to-cath-lab time should be less than 45 minutes.^22^ The ECG-to-cath-lab time in the current study was shorter than the time reported by Tungsubutra and Ngoenjan among patients in Thailand (93 minutes).^16^

There are many potential explanations for the delayed ECG-to-cath-lab time in the current study. The first explanation is that delays in management decisions resulted in delayed activation.^8^ The emergency physicians, in the two selected hospitals, do not have the privilege of activating the cath lab; instead, they are required to wait for the senior cardiologist to confirm the STEMI diagnosis. The second explanation is a delay in obtaining informed consent. The patient could take a long time to provide informed consent. Borden *et al*. reported that Asian people generally took a long time to sign the informed consent.^23^ The Omani cultural practice of obtaining informed PPCI consent is quite different due to cultural considerations; for example, the doctors must wait for a male relative to sign the informed consent for female patients. Moreover, language barriers also play a significant role in delaying informed consent. Future studies are needed to explore the impact of cultural delays in obtaining informed consent on the DTB time.

The third explanation is the limited number of cath labs. In SQUH, there is only one cath lab, and in the Royal Hospital, there are 5 cath labs in the cardiac centre and one in the main hospital building. As a result, if the cath labs were occupied with an elective case, significant delays would arise. When this occurs, the ED staff is told to keep the patient diagnosed with STEMI in the ED until the cath lab is ready. Time spent within the ED and transferring to the cath lab is considered to be the largest component of DTB time. The in-house nursing staff can reduce DTB time by shifting the patient immediately to the cath lab.^24^

The present study showed that the mean time of the cath lab to the balloon was 72.89 ± 42.63 minutes longer than the recommended time of 15 minutes.^22^ The cath-lab-to-balloon time in this study was longer compared to the study done by Zamani *et al*.^8^ They found that the cath lab-to-balloon time was 15 minutes in both the delayed and non-delayed DTB groups. In the cath lab, a delay could occur in the patient handover between the ED and cath lab nursing staff especially, for complicated and unstable patients.^17^^,^^18^ Other possible factors are the patient’s presenting status, comorbidities, procedure characteristics and the number of involved blood vessels.^23^ Future observational studies are needed to explore the factors contributing to the delayed catheterisation to balloon time.

The study’s findings demonstrate that patients who presented in the ED with atypical symptoms of STEMI were 3 times more likely to have delayed DTB times compared to patients presenting with typical symptoms. This is in line with the findings of other studies.^17^^,^^23^^,^^24^ This indicates that the absence of chest pain, which is the typical presenting symptom in patients with STEMI, makes the recognition and identification of such cases more challenging. Therefore, when patients present with atypical symptoms, it causes a delay in the overall DTB time because of a delay in obtaining the ECG which slows the diagnosis.^20^^,^^23^

Identifying patients who are more likely to present with atypical symptoms is critical to time and management. The current study showed that the main characteristics of patients who presented with atypical symptoms were that they were elderly or had diabetes. This finding is consistent with those of other studies.^17^^,^^18^ The gender of the patients being female was also another factor significantly associated with atypical presenting symptoms. In the current study, just over half (53%) of the female patients presented with atypical symptoms. Approximately 90% of the females who presented with atypical symptoms had a delayed DTB time. This resembles the findings of previous studies, which found that females had long DTB times due to atypical symptoms.^21^^,^^25^^,^^26^ It is imperative that ED physicians or triage personnel maintain a high level of suspicion to prevent delays in door-to-ECG times caused by gender and age disparities.

The current study findings also demonstrated that when patients presented with atypical symptoms, nurses tended to assign them to low severity triage levels. The current study showed that out of the total study sample, 192 patients (45.1%) were triaged in the low severity group (levels III, IV or V). More specifically, 4 STEMI patients were assigned to triage level IV (less urgent) and 2 to triage level V (i.e. the non-urgent category). In the study sample, 77 patients (40.1%) who were triaged in the low severity category presented with atypical symptoms. Of these, 69 patients (89.6%) had a delayed DTB time. This indicates that triage nurses were unable to identify the STEMI cases because of the atypical presenting symptoms. Hence, they assigned the patients to less severe triaging levels. Zamani *et al*. reported similar findings.^8^ Assigning qualified nurses to triage and providing them with appropriate training to recognise the symptoms of STEMI are recommended strategies to improve the quality of care.

The current study found that patients presenting after working hours were 2 times more likely to have a delayed DTB time compared with patients presenting to the ED during working hours. In the current study, more than half the study sample (n = 327, 76.8%) presented to the hospital after working hours. The DTB time was 16 minutes longer among patients presenting after working hours compared with patients who presented during regular working hours. This is consistent with the findings of Sorita *et al*. who found that DTB times were longer in patients who presented during off-hours by 14.8 minutes compared to the patients who presented during working hours.^12^

The current study finding was consistent with other studies in that patients with STEMI who presented to the ED during weekends, public holidays and off-hours had longer DTB times compared to those presenting during weekdays and regular working hours.^18^^,^^27^ Several researchers have explained that the delayed DTB time during off-hours is a result of having an insufficient number of on-call cardiologists and support staff for the cardiac cath lab during off-hours. ^12^^,^^27^ This may explain the long response time for the cardiac cath lab.

Reflecting on the clinical practices at the current study sites, the number of ED staff was equal during regular- and off-hours. However, there were fewer cath lab technicians during off-hours. More specifically, during off-hours, both hospitals followed the in-home on-call system. More time could be wasted when the cardiology on-call doctor in the hospital must call members of the on-call cath lab team, such as the cardiology consultant and cath lab technician, to come prepare the cath lab for receiving patients. A patient would be shifted from the ED only after the cath lab team was ready. In some cases, the staff’s commuting distance from the hospitals may affect the laboratory team’s reaction time to prepare the lab. The international standard is for staff to arrive within the recommended 20–30-minute time frame.^28^ Several researchers recommend that the patient be ready in the laboratory when the staff arrives at the hospital to minimise delays caused by long travel times during off-hours.^29^

Hospital policies must implement effective strategies to reduce DTB time and address factors responsible for the delay. The clinical indications in the triage policy for taking the ECG must be expanded, especially for suspected MI cases and for patients who are elderly, female or diabetic and who present with atypical STEMI symptoms. In addition, starting an in-house on-call system for cath lab staff during off-hours can ensure rapid reaction time and timely treatment.^30^ These steps will help identify and manage patients with STEMI, who present to the ED with atypical symptoms and patients who present during off-hours.

In addition, the care manager can conduct a regular auditing system that will help ensure adherence to the recommended guidelines in the ED and cardiology units. The evidence showed that care managers played a vital role in communicating the conditions of patients with heart failure to the interdisciplinary team.^27^^,^^31^ The care manager can communicate the audit results to all involved stakeholders to reduce the delay and find effective practical solutions.

The study has some limitations that should be acknowledged. First, most of the data were taken from nursing and medical notes, which may have been subject to entry errors. Second, this was a cross-sectional retrospective study; hence, assessing the causes of factors leading to delayed DTB times was not possible. Finally, because the study used a retrospective design, several key variables were not assessed because they were unavailable; for example, no data were recorded regarding the patient load in the ED and the time of the cardiologist’s arrival at the ED.

## Conclusion

Performing timely DTB is critical to ensuring safe practices. To meet the DTB time recommendation, it is important to have an effective on-call system that can ensure timely lab activations and transfer of patients. Moreover, there is a need to expand the triage protocol, especially the chief complaints about STEMI symptoms, by including atypical presenting symptoms. Conducting regular training sessions for ED staff is recommended to enhance their awareness of atypical STEMI symptoms and reduce potential DTB delays. The current study results could serve as a baseline for future studies and inform strategies for improving quality of care.

## Figures and Tables

**Table 1:** Characteristics of patients who underwent primary percutaneous interventions at 2 local hospitals in Muscat, Oman (N = 426).

Variable	n (%)			χ^2^	*P* value
	Total population	Non-delayed	Delayed		
**Age in years**					
≤55	199 (46.7)	37 (53.6)	162 (45.4)	1.579	0.209
>55	227 (53.3)	32 (46.4)	195 (54.6)		
Nationality					
Omani	324 (76.1)	50 (72.5)	274 (76.8)	0.584	0.445
Non-Omani	102 (23.9)	19 (27.5)	83 (23.2)		
**Gender**					
Male	348 (81.7)	57 (82.6)	291 (81.5)	0.046	0.829
Female	78 (18.3)	12 (17.4)	66 (18.5)		
**Family history of cardiac disease**					
No	258 (60.6)	40 (58 (	218 (61.1)	0.630	0.362
Yes	168 (39.4)	29 (42 )	139 (38.9)		
**Triage level**					
More severe (levels I & II)	234 (54.9)	39 (56.5)	195 (54.6)	0.084	0.772
Less severe (levels III, IV & V)	192 (45.1)	30 (43.5)	162 (45.4)		
**Initial troponin level**					
Positive	384 (89.9)	61 (88.4)	322 (90.2)	0.204	0.651
Negative	43 (10.1)	8 (11.6)	35 (9.8)		
**History of diabetes**					
No	232 (54.5)	40 (58.0)	192 (53.8)	0.409	0.522
Yes	194 (45.5)	29 (42.0)	165 (46.2)		
**History of hypertension**					
No	223 (52.3)	41 (59.4)	182 (51.0)	1.651	0.199
Yes	203 (47.7)	28 (40.6)	175 (49.0)		
**History of dyslipidaemia**					
No	364 (85.4)	62 (89.9)	302 (84.6)	1.287	0.257
Yes	62 (14.6)	7 (10.1)	55 (15.4)		
**History of heart failure**					
No	419 (98.4)	69 (100)	350 (98.0)	0.241	0.287
Yes	7 (1.6)	0 (0)	7 (2.0)		
**History of previous MI**					
No	366 (85.9)	60 (88.4)	305 (85.4)	0.422	0.516
Yes	60 (14.1)	8 (11.6)	52 (14.6)		
**History of previous PCI**					
No	366 (85.9)	61 (88.4)	305 (85.4)	0.705	0.332
Yes	60 (14.1)	8 (11.6)	52 (14.6)		
**History of previous CABG**					
No	415 (97.4)	67 (97.1)	348 (97.5)	0.033	0.856
Yes	11 (2.6)	2 (2.9)	9 (2.5)		
**Smoking status**					
No	307 (72.1)	53 (76.8)	254 (71.1)	0.921	0.337
Yes	119 (27.9)	16 (23.2)	103 (28.9)		
**Presenting status**					
Stable	386 (89.7)	60 (87)	322 (90.2)		
Cardiogenic shock	19 (4.5)	4 (5.8)	15 (4.2)	0.664*	0.717
Cardiac arrest	25 (5.9)	5 (7.2)	20 (5.6)		
**Presenting symptoms**					
Typical symptoms	302 (70.9)	59 (85.5)	243 (68.1)	8.523	0.005
Atypical symptoms	124 (29.1)	10 (14.5)	114 (31.9)		
**Presenting time**					
Regular	99 (23.82)	25 (36.2)	74 (20.7)	7.791	0.005
Off-hours	327 (76.8)	44 (63.8)	283 (79.3)		

MI = myocardial infarction; PCI = percutaneous coronary interventions; CABG = coronary artery bypass graft.

* Using Fisher’s exact test.

**Table 2:** Distribution of cases with delayed and non-delayed door-to-balloon time across time intervals in minutes

Time interval	Median (IQR)			
	Total population	Non-delayed	Delayed	*P* value
**Door to ECG**	12.00 (5–37)	6.00 (4.50–18)	15.00 (5–45)	0.001
**ECG to cath lab**	78.00 (53–98.25)	40.00 (74.50–20)	82.00 (85–122.50)	<0.001
**Cath lab to balloon**	70.00 (44–90.25)	55.00 (25–78)	75.00 (54–94)	0.020
**Door to balloon**	142.00 (110–190)	75.00 (67–83)	150.00 (128–150)	<0.001

IQR = interquartile range; ECG = electrocardiogram; cath lab = catheterisation laboratory.

**Table 3:** Distribution of door-to-balloon time interval in minutes across study settings

Time interval	Median (IQR)		*P* value
	Hospital A	Hospital B	
**Door to ECG**	10.00 (3–22.50)	25.50 (10–62.25)	<0.001
**ECG to cath lab**	81.00 (60–123.75)	74.00 (35.50–88.25)	0.004
**Cath lab to balloon**	75.00 (50–92.75)	65.00 (35–87.25)	0.028
**Door to balloon**	145.50 (110–193)	136.50 (87.25–108)	0.044

IQR = interquartile range; ECG = electrocardiogram; cath lab = catheterisation laboratory.

**Table 4:** Factors associated with delayed door-to-balloon time (N = 426).

Variable	B	SE	Wald	Adjusted OR (95% CI)	*P* value
**Age (reference: 25–49 years)**	0.176	0.290	0.369	1.193 (0.675–2.105)	0.544
**Female versus male**	−0.226	0.391	0.335	0.797 (0.731–1.715)	0.563
**Negative vs. positive troponin level**	−0.096	0.439	0.048	0.908 (0.385–2.145)	0.826
**H/O diabetes**	0.082	0.305	0.073	1.086 (0.597–1.973)	0.787
**H/O hypertension**	−0.261	0.312	0.698	0.770 (0.418–1.421)	0.403
**H/O dyslipidaemia**	−0.359	0.457	0.615	0.698 (0.285–1.712)	0.433
**H/O previous MI**	−0.233	0.468	0.226	0.800 (0.320–2.003)	0.634
**H/O previous CABG**	0.425	0.903	0.221	1.529 (0.260–8.979)	0.638
**Smokers**	−0.204	0.332	0.377	0.816 (0.425–1.564)	0.539
**Presenting time (off-hours vs. regular working hours)**	0.829	0.295	7.877	2.291 (1.284–4.087)	0.005
**Presenting symptoms (atypical vs. typical)**	1.100	0.386	8.113	3.003 (1.409–6.400)	0.004
**Presenting status (non-stable vs. stable)**	−0.278	0.422	0.435	0.757 (0.331–1.731)	0.509
**Triage level (less to more severe)**	−0.164	0.284	0.333	0.849 (0.487–1.481)	0.564

OR = odds ratio; CI = confidence interval; H/O = history of; MI = myocardial infarction; CABG = coronary artery bypass graft.
